# Supplementary figures and images for: Identification and functional analysis of the CorA/MGT/MRS2-type magnesium transporter in banana
Source: PLoS One. 2020 Oct 1;15(10):e0239058. doi: 10.1371/journal.pone.0239058 (PMC7529347; doi:10.1371/journal.pone.0239058)

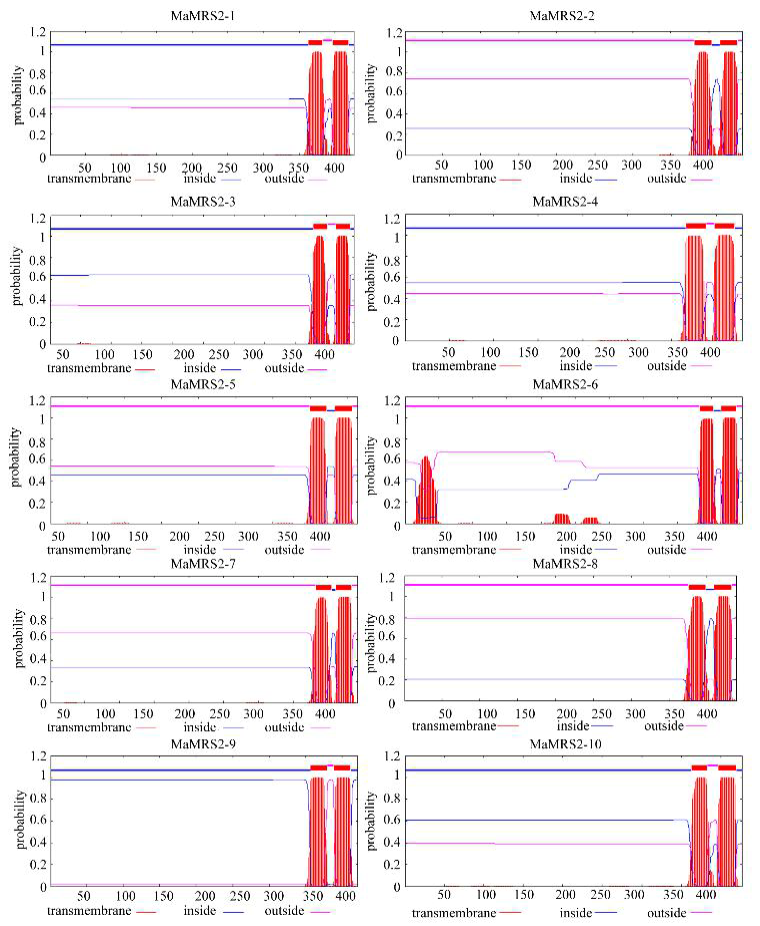

Supplement: S1 Fig — (TIF) [file pone.0239058.s001.tif]

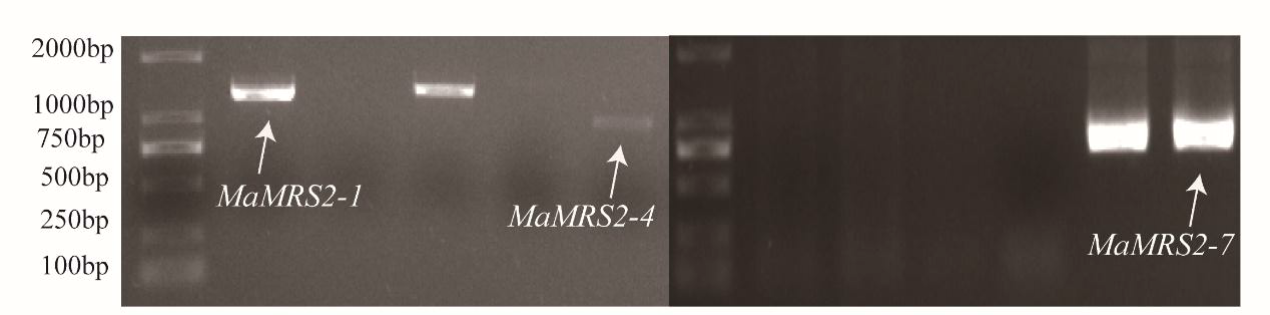

Supplement: S2 Fig — The root or leaf cDNAs were used as template. The RT-PCR was performed 35 cycles using gene specific-primer pairs. (TIF) [file pone.0239058.s002.tif]
