# Supplementary material for: Identification and functional analysis of the CorA/MGT/MRS2-type magnesium transporter in banana
Source: PLoS One. 2020 Oct 1;15(10):e0239058. doi: 10.1371/journal.pone.0239058 (PMC7529347; doi:10.1371/journal.pone.0239058)
Supplement: S1 Table — (PDF) [file pone.0239058.s003.pdf]

Table S1 Ka/Ks of MaMRS2 genes

Gene pairs				
Gene1	Gene 2	Ka	Ks	Ka/Ks
MaMRS2-2	MaMRS2-8	0.08	0.36	0.21
MaMRS2-2	MaMRS2-6	1.11	ND	
MaMRS2-2	MaMRS2-10	0.51	1.94	0.26
MaMRS2-3	MaMRS2-6	0.94	ND	NaN
MaMRS2-4	MaMRS2-8	0.59	2.35	0.25
MaMRS2-4	MaMRS2-6	0.92	ND	
MaMRS2-4	MaMRS2-10	0.51	ND	
MaMRS2-7	MaMRS2-5	0.54	2.92	0.19
MaMRS2-5	MaMRS2-9	0.51	3.02	0.17
MaMRS2-7	MaMRS2-6	1.12	ND	
MaMRS2-8	MaMRS2-6	1.04	ND	
MaMRS2-7	MaMRS2-10	0.44	ND	

ND： not detected
