# Supplementary material for: Identification and functional analysis of the CorA/MGT/MRS2-type magnesium transporter in banana
Source: PLoS One. 2020 Oct 1;15(10):e0239058. doi: 10.1371/journal.pone.0239058 (PMC7529347; doi:10.1371/journal.pone.0239058)
Supplement: S2 Table — (PDF) [file pone.0239058.s004.pdf]

Table S2 PCR primers used for semi-quantitative RT-PCR and qRT-PCR analysis

Gene		Sequence
MaMRS2-1	forward	5'-TACCTGCCGTTCGAGTTTCG-3'
	reverse	5'-CTCTCCGAGTCAAAGCGACA-3'
MaMRS2-2	forward	5'-TGTCGTGCTTGTAGGTCTATTTGG-3'
	reverse	5'-CCTGATTTATAAGCTAGCACGGAG-3'
MaMRS2-3	forward	5'-TTGCTTGGTGTAAGCGCAGG-3'
	reverse	5'-GCAGCAGCCACATCCAATAC-3'
MaMRS2-4	forward	5'-ATGAATATCCCCTGCCAGTTATATG-3'
	reverse	5'-TTGCTGTGAAATTATTTGCTCTGAC-3'
MaMRS2-5	forward	5'-ATGGCGTGGTAAACCCTAGC-3'
	reverse	5'-CAGAAGCCGGAAGTCTCTGG-3'
MaMRS2-6	forward	5'-TTGGCATGAACTTGAAATCCTAC-3'
	reverse	5'-TGTGATCCCTATCATGTCCCTGT-3'
MaMRS2-7	forward	5'-ATGGCGCGGGAAGCG-3'
	reverse	5'-TCATGACCCAATAAGACCTTTGT-3'
MaMRS2-8	forward	5'-ATCCTCTATGTTCTAGCAATTGGGT-3'
	reverse	5'-TGAGCTTGGAGTGTCGTCAATAA-3'
MaMRS2-9	forward	5'-ATAGGGTCCGCAAGCTGAAG-3'
	reverse	5'-GCCGAGTCAACCAAACATGG-3'
MaMRS2-10	forward	5'-TCTAAAAGGCTCGAGAAGGCTTT-3'
	reverse	5'-AGCTGAATGTTAATGAAATCCTCTG-3'
