# Supplementary material for: Identification and functional analysis of the CorA/MGT/MRS2-type magnesium transporter in banana
Source: PLoS One. 2020 Oct 1;15(10):e0239058. doi: 10.1371/journal.pone.0239058 (PMC7529347; doi:10.1371/journal.pone.0239058)
Supplement: S3 Table — (PDF) [file pone.0239058.s005.pdf]

Table S3 PCR primers used for complete CDS amplification

gene		Sequence
MaMRS2-1	Forward	5'-ATGGCGGAGCTCAGAGAACG-3'
	Reverse	5'-TTATAAGGGCATTAATCTTCTATACTTGAAA-3'
MaMRS2-4	Forward	5'-CTGGATGAGGTTCGATCGCGCC-3'
	Reverse	5'-CATTTAAGACCCAAGTAATTTC-3'
MaMRS2-7	Forward	5'-GATTCTGTTCGATTCCAGCGGGG-3'
	Reverse	5'-ATCCCTAAGCAAAACCTCGTCCGC-3'
