# Supplementary material for: Identification and functional analysis of the CorA/MGT/MRS2-type magnesium transporter in banana
Source: PLoS One. 2020 Oct 1;15(10):e0239058. doi: 10.1371/journal.pone.0239058 (PMC7529347; doi:10.1371/journal.pone.0239058)
Supplement: S1 Test — (PDF) [file pone.0239058.s006.pdf]

Test 1 The nucleotide sequences of the three *MaMRS2* genes from sequencing

>MaMRS2-1

CCGAACTGACGTATACGACTCACTATAGGGCGACATATGATCGATGATATCCCATGGGC
GGCCGCCTGCAGACCAGGTCTTTATAAGGGCATTAATCTTCTATACTTGAAATAATACAG
GAACAAAGAGAAAATGGTTACACCGCTTACTCCAGTGATCATTAGCACCCACTGGAAT
GCAGATGGGGCTTTAAATAAGGCTATCTCAAAGTTCATGCCAAATATTCCTGCAACAAC
CCCGAATATGGTAACAACAAACGTCGCTGTCGTTAGTAGCAACTCAAACTGGATCAATT
GATTCCGGACATTATCCAGCTGGATGTTGATGAAATCCTCTGTGTCATCAATATACTCCT
TCAGTGAAGTCAGCTTATTCAGGGTGCTATCAATGACCACAAAGTAAGCCTCTAACAA
CATTTCCAACTCCTCTATGTTCTCGGTAGTGCTGTCAGAACATTTTGTACTCTCATATCT
GCTCCTCGAAAGGCTTAAGGTCTTCTCGAGCTTCCTAGATTCTGGTGGTGAAACAGGT
GAAACTGGAGAAGAAACTGATACTCCATCCCCAGCAGAACTGGACCCATGTAGAGATT
GATCACCATCAAGTGATGAATCCATATGCATTTTCTTCTCTGTGAGACACATTTCGGCCA
TGTCACCATCATCATCCATCAATTGCTCAATTTCATCTCTAACCTTTTGAACTCTCCGAG
TCAAAGCGACAAGCCTGCTTTTTAGGCGACGCACCCGTTCCAAGTTAAGAGTACTGAT
CTTTGAGGTTAATTCATCTAACAAGGGGTATGCCTCTATTTCTAGATCCGAAGCCTGAGC
GTCCAGGAAGGTGCAGGCGGCCTCGAGCGCGACCTCGAGGGCTCGAAACTCGAACG
GCAGGTAATCAGGCGATGGCGTGCCGCCGTCGAGGGCGTCTCTCGTGCCGGCAGCGA
GGCGGCGCTGCAACTCAACAACGTACTGCAGGACGTAGCCGTCGAAGGAGTTGAAGA
GGAGCACCTCGTCGGCGGTGATGATGCATCGGATCTTCTCGAGGTTGACGACGATCGC
CCGCTCCCGGCCGAGGATGGTGGAGGGGTAGATGAATACGGGGTCGAGGAGGCGAAG
GTCACGGGCGGGGAGGTCGCAGCGCCGCATCATGGTGAACTTATCGACCTCGATCACC
TGCGATCCGCCCGTGGCTGCGTCGACGCGGATCCACGAGCGGAGGCTCTGCCCCCGCT
TCTTGAGCTCGGAAAAGTCGACACCTTGGAAGAGGGGGCGGCAGCAGGGGGGGGAC
TCGCGGGCGGCTGTCGAGGGGAGCGGGACAGCGGCGACGGAGGGCTTTCGTGGGAG
GAGCCGTTCTCTGAGCTCCGCCATAAGACTGGAGATCTGGATCCCTCGAGTCTAGAGT
CGACCTGCAGGCATGCATGCAATC

>MaMRS2-4

GCGTAGTTGTCGTATACGACTCACTATAGGGCGACATATGATCGATGATATCCCATGGGC
GGCCGCCTGCAGACCAGGTCTCTGGATGAGGTTCGATCGCGCCGGGCAGTCGGAGGT
CCTGGAACTCGACAAGAGCGCCATCATCAAGCGGGCGGGGATTCCTCCCCGCGACCTT
AGGATTCTTGGACCCGTCTTCTCCCATTCGTCGAACATCCTCGCCAGGGAAAAGGCTAT
GGTTGTCAATCTAGAATTCATAAAGGCCATTGTTACTGCTGATGAAGTGCTAATACTGGA
TCCTCTGTGTCAAGAAGTTCTCCCTTTTGTAGATCAGTTAAGGCAACAACTCCCTTTGA
AGAGTCCTTTCAGGGTCGATGATCCTAATCTTGATAAACAAAGCAAAGATAAACATGCA
ACTGGTGAAGAGTGGACAAAAATAAATGAGGCAGCTGAAAGTGAACGTGAACTACCA
TTTGAGTTTCAGGTGCTTGAGATTGCACTAGAAGTTGTTTGTTCATATTTGGACTGTAG
CGTCTCTGATCTTGAGAAAAATGCATACCCTGTGCTTGATGAATTAGCCATGAATGTTA
GCACCAAGAATCTTGAGCGTGTGCGGAGTCTGAAAAGCAATCTTACTCGTTTGCTTGC
ACATATTCAGAAGGTTAGGGATGAAATTGAACACCTTTTAGATGACAACGAGGATATGG
CCCATCTATATTTAACAAGGAAGCAAATCCAGAATCAGCAGTTTGAAGCTTTGATAGCC
TCTGGGGCTTCAAATAGCATTGTTGCTGCAGGACCAAACCTCGCAAGACTTGGCTCTA
ATTTAAATTGTAGCGCAAGCATTATTTCTAGTATCTATGCTGATGATAATGATGTGGAGGA
TTTGGAGATGTTACTTGAAGCTTATTTCATGCAGCTTGATGGAATGCGCAACAAAATTT

TGTCGGTGAGGGAATATATTGATGATACAGAAGACTACGTCAACATCCAGCTTGATAAC
CAGCGAAATGAACTTATTCAACTCCAGTTGGTATTAACCATTGCATCATTCGGCATAGCT
GTAGACACCCTTATCGTAGGTGCATTTGCAATGAATATCCCCTGCCAGTTATATGATATTA
ACCACATCTTTACTCCCTTTGTCGGAGGCACATCAGGAGGCTGGGTCCTGATCACCTTG
TTCATGCTAGCATATGCCAGGTGGAAGAAATTACTTGGGTCTTAAATGAAGACTGGAGA
TCTGGATCCCTCGAGTCTAGAGTCGACCTGCAGGCATGCATGCTGTG

>MaMRS2-7

CCGGGGTAGGTGATTCGAGCTCGGTACCGTAATACGACTCACTATAGGGCGACATATGA
TCGATGATATCCCATGGGCGGCCGCCTGCAGACCAGGTCTATGGCGCGGGAAGCGCTG
GCAGTGCCCGCGGAGGCGCAGGCCGCGCTCAAGAAGAAGACGGCGGCCGCGAGGAG
CTGGATTCTGTTCGATTCCAGCGGGGAAGGGACAATCTTGGACGTCGACAAGTACGCC
ATCATGCACCGGGTTCAGATCCACGCGCGCGACCTCAGGATCCTGGACCCCCTCCTCT
CGTACCCTTCCACGATTTTGGGACGCGAGCGAGCCATCGTTCTCAATCTTGAGCACATT
AAAGCGATCATCACCGCGGACGAGGTTTTGCTTAGGGATCCCGCAGAAGAAAATGTTG
TTCCAATTGTGGAAGAGCTTCAAAGACGATTACCACTGGCAAACGTTGTCAATAATGC
ACATGGAGAGGGGAGAGAGAATGTTACTGGACAACATGACGTGGAAGCTGTTGAAGA
AGATGAATCTCCCTTTGAGTTTCGAGCCCTGGAAGTTGCTTTAGAAGCTATTTGTAGCT
ATCTTGATGCACGCACCAGTGAACTAGAGACTGCTGCTTACCCGGCCTTAGATGAGCT
GACTTCCAAGATTAGCAGCCGTAACTTGGATCGAGTGCGTAAGTTGAAGAGTGCCATG
ACTAGGTTGACTGCTCGTGTCCAAAAGGTAAGGGATGAGCTTGAACAGTTATTGGATG
ACGATGATGATATGGCAGATCTTTACTTGTCAAGGAAACTGGCTGGAGCATCCTCTCCT
GTCAGCGGTTCTAGTATGCCTAACTGGCTCCCTGCTTCACCAACAATTGGATCTAGGAT
ATCCAGAGCAAGCAGGGCAAGTGCAGCAACTATACATGGAAATGAGAATGATGTTGAG
GAGCTGGAAATGTTACTCGAGGCATACTTCATGCAAATTGATGGCACATTGAACAAGTT
GACTACTCTACGCGAATATATTGATGACACAGAGGACTATATCAATATTCAGCTTGACAA
CCACCGAAACCAACTTATTCAGCTAGAGCTGTTCTTGAGTTCTGGCACAGTTTCCCTCT
CAATATATTCACTGGTTGCTGGGATATTTGGAATGAACATACCGTATTCATGGAATGATG
ACCATGGACATGTATTCAAATGGGTGGTCATTTTGTCGGGACTTATCAGTGGCTTCCTG
TTCATTTTCATAATTGCTTATGCTCGCCACAAAGGTCTTATTGGGTCATGAAGACTGGAG
ATCTGGATCCCTCGAGTCTAGAGTCGACCTGCAGGCATGCAAGCTGGCGTAATCCATG
GTC
