# Supplementary material for: Identification and functional analysis of the CorA/MGT/MRS2-type magnesium transporter in banana
Source: PLoS One. 2020 Oct 1;15(10):e0239058. doi: 10.1371/journal.pone.0239058 (PMC7529347; doi:10.1371/journal.pone.0239058)
Supplement: S1 Raw images — (PDF) [file pone.0239058.s007.pdf]

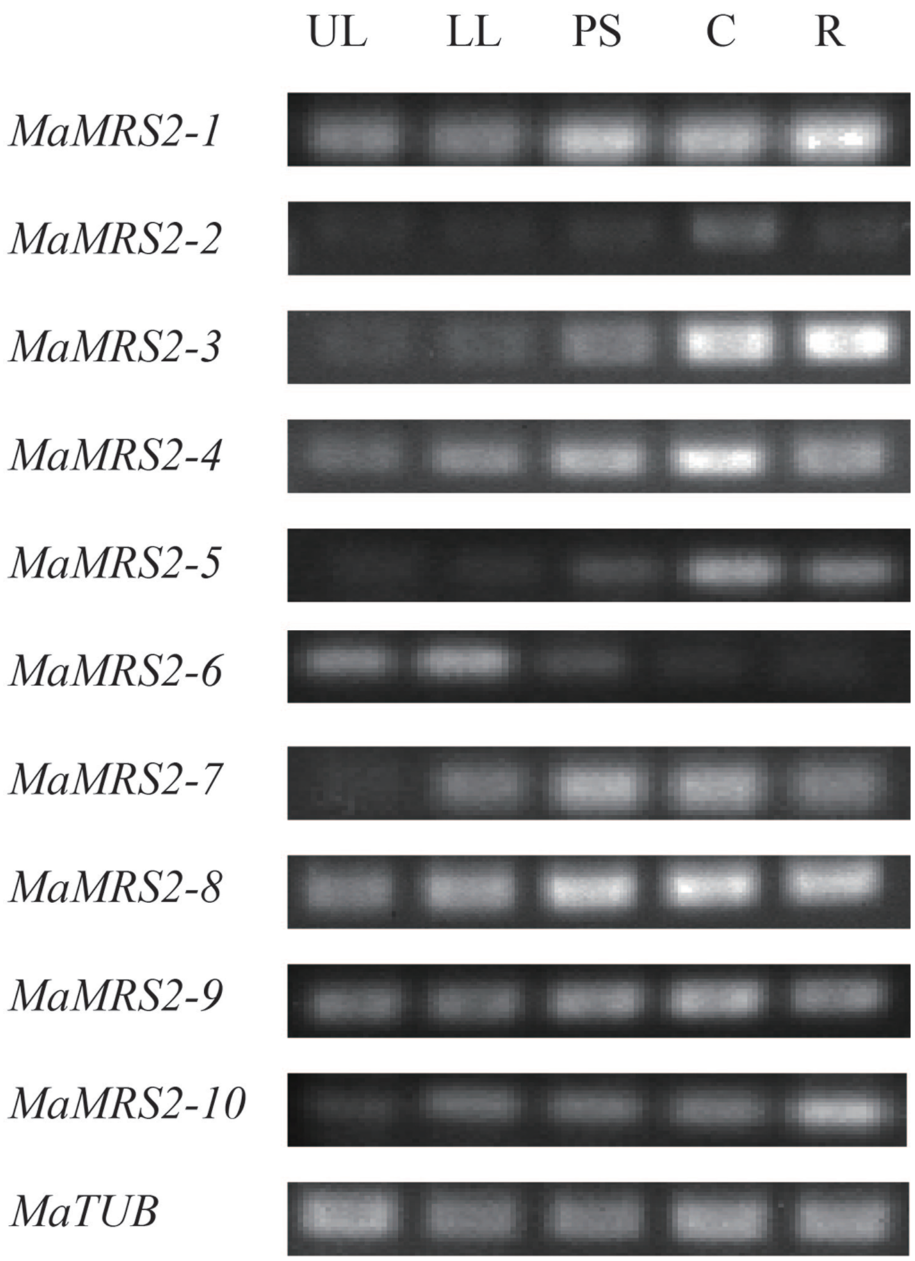

Fig.7 Expression of 10 *MaMRS2* genes in different tissues of banana cultivar Baxijiao seedlings.
Using gel electrophoresis imaging system to generate images. upload order is No.9.

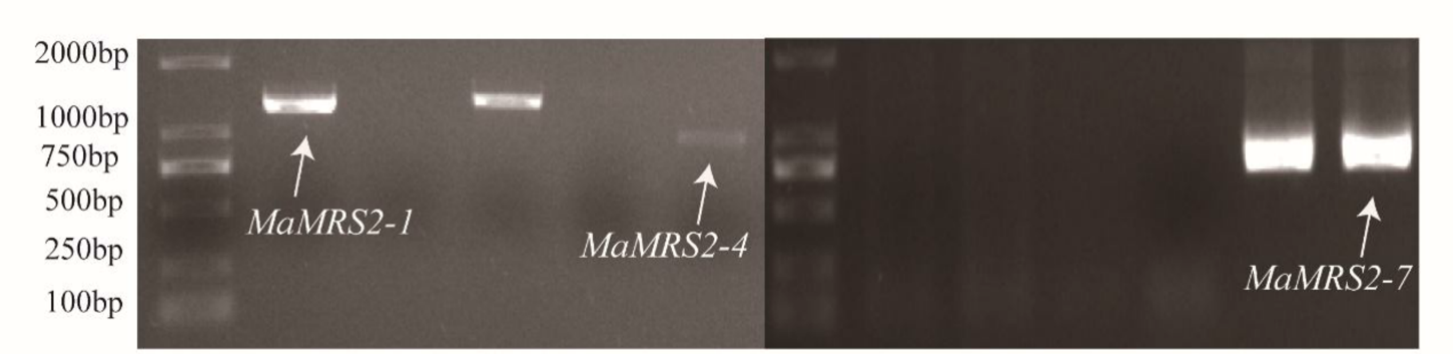

Fig.S2 RT-PCR amplification of the entire ORF of Three *MaMRS2* genes.
Using gel electrophoresis imaging system to generate images. upload order is No.12.
