# Supplementary material for: Identification and functional analysis of the CorA/MGT/MRS2-type magnesium transporter in banana
Source: PLoS One. 2020 Oct 1;15(10):e0239058. doi: 10.1371/journal.pone.0239058 (PMC7529347; doi:10.1371/journal.pone.0239058)
Supplement: Attachment — Submitted filename: Response to Reviewers.docx [file pone.0239058.s008.docx]

Reviewer #1: 1. The author said that these genes were not only involved in Mg uptake and transport, but also participated in Mg allocation among banana’s root, pseudo stem, corm, and leaf components，which required more biological experiments to support it

Response： the original sentences“The contrasting expression pattern of different Mg transporter genes among differing tissues under Mg deficiency (Fig. 8) indicated these genes were not only involved in Mg uptake and transport, but also participated in Mg allocation among banana’s root, pseudo stem, corm, and leaf components”in page 7 line 21 - 24 changed to:

“The contrasting expression pattern of different Mg transporter genes among differing tissues under Mg deficiency (Fig. 8) indicated these genes is probably not only involved in Mg uptake and transport, but also participated in Mg allocation among different tissues.”in page 7 line 13 – 16 in the revised version.

2. need adding bilogical stastics for q-PCR data

Response：We analyzed the significance of q-PCR data, as shown in Figure 8.

3. DISCUSSION should be rewritten.

Response: We changed the 1-8 paragraphs in the original DISCUSSION to the 1-3 paragraphs in the revised version.

4. writting and orgnization should be further improved.

Response: We carefully read the manuscript and improved the writting in the revised version.

In the original version in page 1 line 10 – 11: “and the CorA/MGT/MRS2 family proteins are considered vital to this process.” was change to: “especially CorA/MGT/MRS2 family proteins, played a vital role in regulating Mg content in plant cells” in page 1 line 10 -11;

In the original version in page 1 line 12 – 13: “much less is known about it for tropical crops.” was change to: “the relevant information is scarce in tropical crops.” in page 1 line 11 - 12;

In the original version in page 1 line 15: “chromosomes” was change to: “chromosome” in page 1 line 15;

In the original version in page 1 line 18 – 19: “branches of the phylogenetic tree” was change to: “phylogenetic” in page 1 line 18;

In the original version in page 1 line 22 – 28: “Specifically, among the MaMRS2 genes, MaMRS2-2 was expressed only in the corm, and MaMRS2-6 was most expressed in the leaves. This result was confirmed by real-time PCR analysis which uncovered differential MaMRS2 gene expression under Mg2+ deficiency conditions, in that MaMRS2-5 and MaMRS2-7 were both up-regulated in stems, whereas MaMRS2-1 and MaMRS2-10 were down-regulated in roots, stems, and leaves. These results will contribute to the further study of Mg transporters in banana.”

was change to:

“The result was confirmed by real-time PCR analysis, in addition to tissue specific expression, expression differences among MaMRS2 members was also observed under Mg deficiency conditions. These results showed that Mg transporters may play a versatile role in banana growth and development, and our work will shed light on the functional analysis of Mg transporters in banana.” in page 1 line 21 - 26;

In the original version in page 2 line 1 - 4 “the concentration of Mg in metabolic pools, such as the cytoplasm and chloroplast, are strictly regulated, for which magnesium transporter (MGT) plays a vital role in maintaining the equilibrium and homeostasis of Mg2+ in plants [6, 7].” changed to “The concentration of Mg in metabolic pools, such as the cytoplasm and chloroplast, are strictly regulated. And magnesium transporter (MGT) plays a vital role in maintaining the equilibrium and homeostasis of Mg in plants [6, 7].” in page 1 line 37 – 38 and page 2 line 1 -2 in the revised version.

In the original version in page 5 line 24 - 29 “was” changed to “were” in page 5 line 17 – 19 in the revised version.

Reviewer #2: The authors characterized the Magnesium transporter *MaMRS2* protein family. 10 *MaMRS2* genes in banana (*Musa acuminata*) were identified. The physicochemical properties, location on chromosomes, gene structure, cis-acting elements, and replication relationships between these ten members were analyzed. The tissue-specific expression pattern was analyzed. Three genes *MaMRS2-1, MaMRS2-4*, and *MaMRS2-7* were cloned and complemented with triple MGT mutant of Salmonella typhimurium. This study could help in understanding the function of each *MaMRS2* gene in development or stress conditions.

The manuscript is poorly written and needs to be largely reworked.
Response：the original part in page 1 line 13-29 “In this study, 10 *MaMRS2* genes in banana (*Musa acuminata*) were isolated from its sequenced genome and classified into five distinct clades. The physicochemical properties, location on chromosomes, gene structure, cis-acting elements, and duplication relationships between these 10 members were analyzed. Complementary experiments revealed that three MaMRS2 gene members (*MaMRS2-1, MaMRS2-4, MaMRS2-7*), from three distinct branches of the phylogenetic tree, were capable of restoring the function of Mg^2+^ transport in Salmonella typhimurium mutants. Semi-quantitative RT-PCR showed that the 10 members of the *MaMRS2* gene were differentially expressed in the root, pseudo stem, corm, and leaves of banana cultivar ‘Baxijiao’ (*Musa spp. AAA Cavendish*) seedlings. Specifically, among the *MaMRS2* genes, *MaMRS2-2* was expressed only in the corm, and *MaMRS2-6* was most expressed in the leaves. This result was confirmed by real-time PCR analysis which uncovered differential *MaMRS2* gene expression under Mg^2+^ deficiency conditions, in that *MaMRS2-5* and *MaMRS2-7* were both up-regulated in stems, whereas *MaMRS2-1* and *MaMRS2-10* were down-regulated in roots, stems, and leaves. These results should be helpful to further research work on Mg transporters in banana crops.” changed to:

“In this study, 10 *MaMRS2* genes in banana (*Musa acuminata*) were isolated from its genome and classified into five distinct clades. The putative physicochemical properties, chromosome location, gene structure, cis-acting elements, and duplication relationships in between these members were analyzed. Complementary experiments revealed that three *MaMRS2* gene members (*MaMRS2-1, MaMRS2-4, MaMRS2-7*), from three distinct phylogenetic branches, were capable of restoring the function of Mg transport in Salmonella typhimurium mutants. Semi-quantitative RT-PCR showed that *MaMRS2* genes were differentially expressed in banana cultivar ‘Baxijiao’ (*Musa spp. AAA Cavendish*) seedlings. The result was confirmed by real-time PCR analysis, in addition to tissue specific expression, expression differences among *MaMRS2* members was also observed under Mg deficiency conditions. These results showed that Mg transporters may play a versatile role in banana growth and development, and our work will shed light on the functional analysis of Mg transporters in banana.” In page 1 line 13 -29 in the revised version

1. Rewrite the line: These results should be helpful to further research work on Mg transporters in banana crops.

Response：the original “These results should be helpful to further research work on Mg transporters in banana crops” in page 1 line 27 - 29 changed to:

“These results showed that Mg transporters may play a versatile role in banana growth and development, and our work will shed light on the functional analysis of Mg transporters in banana.” in page 1, line 24-26 in the revised version.

2. The introduction is very messy, especially the expression summary of MGTs in different plants. Rewrite the introduction.

Response: we have rewritten the INTRODUCTION.

The original sentence in page 1 line 32 – 33 “ During plant growth and development, magnesium (Mg) is essential and it cannot be substituted[1]” changed to：

“Magnesium (Mg) is essential in plant growth and development, and cannot be substituted[1].” In page 1 line 30 – 31 in the revised version;

The original sentence in page 1 line 34-36 “The major function of Mg in green leaves is forming the central atom of chlorophyll, Mg is also involved in protein synthesis as a bridging element for the aggregation of ribosome subunits [2], and functions as a stabilizer of specific conformation in nucleic acid synthesis [3]” changed to: “Mg is also involved in protein and nucleic acid synthesis, and it acts as a bridging element for the aggregation of ribosome subunits [2] and a conformation stabilizer [3] respectively.” In page 1 line 32 - 34 in the revised version;

The original sentence in page 2 line 1 – 4 “The concentration of Mg in metabolic pools, such as the cytoplasm and chloroplast, are strictly regulated, **for which magnesium transporter (MGT) plays a vital role in maintaining the equilibrium and homeostasis of Mg2+ in plants [6, 7].**” changed to: “The concentration of Mg in metabolic pools, such as the cytoplasm and chloroplast, are strictly regulated. **And magnesium transporter (MGT) plays a vital role in maintaining the equilibrium and homeostasis of Mg in plants [6, 7].**” In page 1 line 37 – 38 and page 2 line 1 – 2 in the revised version;

The expression summary of MGTs in different plants in the original part in page 2 second and third paragraphs changed to page 2 second paragraph in the revised version；

The original sentence in page 3 line 6 – 10 “Imbalanced fertilization practices aggravates the likelihood of Mg deficiency occurring in cultivated banana, such that Mg deficiency is now a major reason for reduced banana fruit yield [30]. Therefore, improving the efficiency of Mg utilization in banana has immediate practical significance. Clearly, MGT plays a vital role in Mg nutrition maintence in plant species.” changed to: “Imbalanced fertilization practices aggravates the likelihood of Mg deficiency, as a result, Mg deficiency is now a major contributor to banana yield reduction [30]. Therefore, improving the efficiency of Mg utilization in banana has immediate practical significance. Clearly, MGT plays a vital role in Mg nutrition maintenance in plants.” in page 2 line 41 – 44 and page 3 line 1 in the revised version;

3. Units are missing: The isoelectric point of the predicted proteins and their corresponding amino acid length ranged respectively from 4.51 to 9.16 (pI) and from 379 to 495 (aa).

Response: We have supplemented the corresponding units “Identification of MRS2 family genes in banana” ,the isoelectric point, the amino acid length and the molecular weight of the predicted proteins ranged from 4.51 to 9.16 (pI), from 379 to 495 (aa), and from 42.57 to 54.83 (kDa)” in Page 3 line 15 in the revised version.

4. Rewrite: Figure 2 shows the phylogenetic tree built for Mg transporter protein sequences from banana, Arabidopsis, rice, maize, and yeast, with yeast Mg transporters as the outgroup.

Response：the original sentence in page 5 line 4 - 6 “Figure 2 shows the phylogenetic tree built for Mg transporter protein sequences from banana, Arabidopsis, rice, maize, and yeast, with yeast Mg transporters as the outgroup” changed to:

“Phylogenetic trees were constructed using MGTs of banana, Arabidopsis, rice, maize and yeast. Among them, MGTs of yeast was selected as the outgroup (Fig. 2)”. In Page 5, line 3 – 4 in the revised version.

5. Chromosomal location and gene duplication: Authors mentioned gene duplication as gene replication. (5 times). There is a difference between the two terms.

Also, in the discussion part: we found evidence for replication relationships that occurred between MaMRS2 family members across chromosomes but no signs of a tandem replication relationship (Fig. 4). This pattern generally exists among other gene family members of banana [32]. It is reasonable to speculate that multiple rounds of whole genome replication events have occurred over evolutionary time in banana.

Response: we modified “replication” in page 5 line 39 = 42 and page 6 line 1 to “duplication” in page 5 line 37 – 42 (5 times) in the revised version.

Also, the original sentence “This pattern generally exists among other gene family members of banana [32]. It is reasonable to speculate that multiple rounds of whole genome replication events have occurred over evolutionary time in banana.” in page 8 line 14 -16 changed to:

“Genomic collinearity showed that duplication relationships occurred between MaMRS2 family members across chromosomes, but no signs of a tandem duplication relationship (Fig. 4), a pattern generally exists among other gene family members of banana” in the revised version.

6. Figure legends are not uploaded properly or incomplete:
Fig. 1 Multiple sequence alignments of MaMSR2 proteins. This alignment was performed using DNAMAN software. The identical, conserved, and less conserved
Fig. 2 Phylogenetic analysis of Arabidopsis, rice, maize, yeast, and banana CorA/MRS2/MGT members. The neighbor-joining tree, which includes 10 MaMRS2
Fig. 3 Phylogenetic relationships (A), gene structure (B), and motif analysis (C) of MaMRS2 family members in banana. The phylogenetic tree was constructed using the
Fig. 4 Chromosomal location and gene duplication of MaMRS2 genes in the banana genome. The MaMRS2 gene is located on multiple chromosomes. The chromosome
Fig. 5 Predicted cis-acting elements in the promoter region of the 10 MaMRS2 genes in banana. Different colors represent cis-acting elements associated with different
Fig. 6 Complementary analysis of the MaMRS2 genes. MM1927 is the wild type, used here as a positive control; the MM281 and
Fig. 7 Expression of 10 MaMRS2 genes in different tissues of banana cultivar ‘Baxijiao’ (Musa spp. AAA Cavendish) seedlings. Explain the Abbreviations.
Fig. 8 Relative expression levels of 10 MaMRS2 genes in different tissues of banana cultivar ‘Baxijiao’ (Musa spp. AAA Cavendish) Explain the Abbreviations.
Response: We have uploaded the complete Figure legends.

Fig.1 Phylogenetic analysis of Arabidopsis, rice, maize, yeast and banana CorA/MRS2/MGT members. The Neighbor-Joining tree, which includes 10 MaMRS2 protein from banana, 11 MRS2/MGT proteins from Arabidopsis, 9 MRS2/MGT proteins from rice, 12 MRS2/MGT proteins from maize and 5 MRS2/MGT proteins from yeast, was constructed using MEGA X. A, B, C, D, E, F, G and H represent the different clades of the MRS2/MGT family in these five species.

Fig.1 Multiple sequence alignments of MaMSR2 proteins. Alignment was performed using DNAMAN software. The identical, conserved and less conserved amino acid residues are indicated by dark, cherry red and cyan background colors, respectively. The conservative GMN motif was indicated and the TM domains are underlined.

Fig.2 Phylogenetic relationships (A), gene structure (B) and motif analysis (C) of MaMRS2 family members. The Phylogenetic tree was constructed using the Neighbor-Joining method with 1000 bootstrap replicates in the MEGA X software. Then the gene structure was performed using GSDS program. MEME program and TBTools were used to illustrate the motif analysis results. Yellow boxes and black lines represent exons and introns, respectively, blue boxes indicate 5’ or 3’ untranslated regions (UTRs) and different motif was painted with different color.

Fig.3 Phylogenetic relationships (A), gene structure (B) and motif analysis (C) of MaMRS2 family members. The Phylogenetic tree was constructed using the Neighbor-Joining method with 1000 bootstrap replicates in the MEGA X software. Then the gene structure was performed using GSDS program. MEME program and TBTools were used to illustrate the motif analysis results. Yellow boxes and black lines represent exons and introns, respectively, blue boxes indicate 5’ or 3’ untranslated regions (UTRs) and different motif was painted with different color.

Fig.4 Chromosomal location and gene duplication of *MaMRS2* genes in the banana genome. The *MaMRS2* gene is located on different chromosomes. The number of chromosomes is shown on the outside, and the different colors represent different chromosomes. The grey region is the collinearity of the banana genome, highlighting the collinearity between the MaMRS2 genes was highlighted with red lines.

Fig.5 Analysis of cis-acting elements of members of the *MaMRS2* gene family. Different colors represent cis-acting elements of different functions.

Fig.6 Complementary analysis of the *MaMRS2* genes. MM1927 was the wild type and used as a positive control, MM281 and MM281 transformed with an empty pTrc99A were used as negative controls. (A) Functional verification on N minimal solid medium containing 20, 10, 5, 2, 1, 0.5, 0.1, 0.01 mM MgSO4. The bacterial was diluted sequentially 10-fold from left to right. (B) Growth curves were performed in N-minimal liquid medium containing 10, 1, 0.5 and 0.01mM MgSO4, and the cell density was monitored at OD600 every 2 hours. Data was an average of three independent experiments, and the different icons in the figure represent the average of three repetitions.

Fig.7 Expression of *MaMRS2* gene in different tissues of Baxijiao seedlings. UL, LL, PS, C and R represent upper leaf, lower leaf, pseudo stem, corm and root respectively.
Fig.8 Relative expression level of MaMRS2 gene in different tissues of Baxijiao seedlings under magnesium deficiency. The CK indicated the value under normal growth conditions as a control, and the -Mg indicated the relative expression level under the complete absence of magnesium ion condition. UL, LL, PS, C and R represent upper leaf, lower leaf, pseudo stem, corm and root respectively.

7. The author mentioned sequences of 7 genes:
Supplementary Test1 The nucleotide sequences of the seven *MaMRS2* genes from sequencing
There are only three sequences >MaMRS2-1, >MaMRS2-4, >MaMRS2-7 as authors clones only three genes.

Response: The original “Supplementary Test1 The nucleotide sequences of the seven *MaMRS2* genes from sequencing” changed to “Supplementary Test1 The nucleotide sequences of the three *MaMRS2* genes from sequencing” see Supplementary test1.

8. The authors did complementation assay with MM281 mutant.
Figure 6A: Why the growth of complemented lines is low in higher concentration of magnesium as compare to Mutant (MaMRS2-4, MaMRS2-7), as Complemented lines are growing fine in lower concentrations.

Response: We speculate that the growth of the transformants has an optimal concentration. the genes from banana may be less efficient under high Mg condition when compared with that from bacteria, while they are similar under low Mg conditions.

Figure 6B: Authors mentioned: The data are shown as the mean ± SD of three biological replicates,
However, no SD is shown on the line charts.

Response: “The data are shown as the mean ± SD of three biological replicates,” in page 12 line 19 changed to “The data are shown as the mean of three biological replicates,” in page 11 line 4 in the revised version.

The growth curve at 0.01 mM Mg^2+^ shows that complementing lines 2-1 and 2-7 are growing better than the wild type, which does not correlate with Figure 6A.

Response: We carefully checked the original data and found that there were errors in the data processing. Now we have corrected the original data. Figure 6B 0.01mm is corrected (please see the revised figure 6B):

9. qRT-PCR analysis of MaMRS2-9 has no error bars; what does it signify? Also, many other samples have no or negligible error bar, which cannot be true for three biological replicates. The authors did not mention technical replicates.

Response: The histogram in Figure 8 is the average of three technical repetitions with 3 biological replicates, we revised it in the new manuscript with error bars added.

Tubulin primers used in the current study are new or from the previous study? If from the previous study needs a reference.

Response: Tubulin primers used in the current study are from the previous study, The reference was in Page 12, line 29 - 31 “The expression level of each *MaMRS2* gene was calculated using the 2^-ΔCt^ method, for which the TUB gene served as the internal reference [56]”. See Reference: 56. Podevin N, Krauss A, Henry I, Swennen R, Remy S. Selection and validation of reference genes for quantitative RT-PCR expression studies of the non-model crop Musa. Mol Breeding. 30(3): 1237-1252.https://doi.org/10.1007/s11032-012-9711-1 PMID: 23024595.

Also, we carefully read our original article and found some mistakes, as follows:

the original sentences in page 6 line 2“The Ka/Ks of five homologous Mg transporters was < 0.3 (Supplementary Table S1);” have changed to “The Ka/Ks of five homologous Mg transporters was less than 0.3 (Supplementary Table S1);”in page 5 line 43-44 in the revised version.

We add the reference of Fig. 1 in page 3 line 24, Fig. 4 in page 8 line 9, Fig. 5 in page 8 line 31 in the revised version.
