# Supplementary material for: Identification and functional analysis of the CorA/MGT/MRS2-type magnesium transporter in banana
Source: PLoS One. 2020 Oct 1;15(10):e0239058. doi: 10.1371/journal.pone.0239058 (PMC7529347; doi:10.1371/journal.pone.0239058)
Supplement: Attachment — Submitted filename: Response to Reviewers.docx [file pone.0239058.s009.docx]

Reviewer #2:

1. Authors may include this response in Discussion:
   Comment8. The authors did complementation assay with MM281 mutant.
   Figure 6A: Why the growth of complemented lines is low in higher concentration of
   magnesium as compare to Mutant (*MaMRS2-4*, *MaMRS2-7*), as Complemented lines
   are growing fine in lower concentrations.
   Response:

In the reviewed manuscript at the end of the second paragraph of the discussion, we added the following:

The complementary lines incorporated with *MaMRS2-4* and *MaMRS2-7* could grow well under low Mg concentration conditions, but they had lower growth rate when compared with the mutant control under solid growth medium with higher Mg concentration (10 mM and 20 mM) (Fig. 6A). These results indicated that both *MaMRS2-4* and *MaMRS2-7*could transport Mg, at the same time, the exogenous Mg transporters from banana might play its role in excessive Mg accumulation and lead to the toxic effect in bacteria under high Mg conditions.

2. Also, we carefully read our original article and found some mistakes, as follows:

The “physicochemical” was changed to “physiochemical” in page 1 line 15；

“was” was changed were” in page 1 line 23;

“isocitrate-lyase” was changed “isocitrate lyase” page 1 line 35;

“RuBP carboxylase” was changed “ribulose bisphosphate carboxylase” in page 2 line 36;

“diversed” was changed “diverse” in page 2 line 35;

“Protiens” was changed “proteins” in page 9 line 6;

“Figure. 6 A” was changed “Fig. 6” in page 9 line 7;

“Ca(NO_3_)·4H_2_O” was changed “Ca(NO_3_)_2_·4H_2_O” in page 11 line 20

“Chen C, Xia R, Chen H, He Y. TBtools, a Toolkit for Biologists integrating various HTS-data handling tools with a user-friendly interface. bioRxiv. 2018: 289660. <https://doi.org/10.1101/289660>.” was changed to “Chen C, Chen H, Zhang Y, et al. TBtools-an integrative toolkit developed for interactive analyses of big biological data[J]. bioRxiv, 2020: 289660. [https://doi.org/10.1016/j.molp.2020.06.009”](https://doi.org/10.1016/j.molp.2020.06.009\”) in page 16 line 15-17.
